# Regional Radiomics Similarity Networks Reveal Distinct Subtypes and Abnormality Patterns in Mild Cognitive Impairment

**DOI:** 10.1002/advs.202104538

**Published:** 2022-01-31

**Authors:** Kun Zhao, Qiang Zheng, Martin Dyrba, Timothy Rittman, Ang Li, Tongtong Che, Pindong Chen, Yuqing Sun, Xiaopeng Kang, Qiongling Li, Bing Liu, Yong Liu, Shuyu Li, for the Alzheimer's Disease Neuroimaging Initiative

**Affiliations:** ^1^ Beijing Advanced Innovation Centre for Biomedical Engineering School of Biological Science and Medical Engineering Beihang University Beijing 100191 China; ^2^ School of Artificial Intelligence Beijing University of Posts and Telecommunications Beijing 100876 China; ^3^ School of Computer and Control Engineering Yantai University Yantai 264005 China; ^4^ German Center for Neurodegenerative Diseases (DZNE) Rostock 18147 Germany; ^5^ Department of Clinical Neurosciences University of Cambridge Cambridge Biomedical Campus Cambridge CB2 0SZ UK; ^6^ State Key Laboratory of Brain and Cognitive Science, Institute of Biophysics Chinese Academy of Sciences Beijing 100101 China; ^7^ Brainnetome Center & National Laboratory of Pattern Recognition Institute of Automation Chinese Academy of Sciences Beijing 100190 China; ^8^ School of Artificial Intelligence University of Chinese Academy of Sciences Chinese Academy of Sciences Beijing 100049 China; ^9^ State Key Laboratory of Cognition Neuroscience & Learning Beijing Normal University Beijing 100875 China

**Keywords:** mild cognitive impairment, progression, regional radiomics similarity network, subtypes

## Abstract

Individuals with mild cognitive impairment (MCI) of different subtypes show distinct alterations in network patterns. The first aim of this study is to identify the subtypes of MCI by employing a regional radiomics similarity network (R2SN). The second aim is to characterize the abnormality patterns associated with the clinical manifestations of each subtype. An individual‐level R2SN is constructed for *N* = 605 normal controls (NCs), *N* = 766 MCI patients, and *N* = 283 Alzheimer's disease (AD) patients. MCI patients’ R2SN profiles are clustered into two subtypes using nonnegative matrix factorization. The patterns of brain alterations, gene expression, and the risk of cognitive decline in each subtype are evaluated. MCI patients are clustered into “similar to the pattern of NCs” (N‐CI, *N* = 252) and “similar to the pattern of AD” (A‐CI, *N* = 514) subgroups. Significant differences are observed between the subtypes with respect to the following: 1) clinical measures; 2) multimodal neuroimaging; 3) the proportion of progression to dementia (61.54% for A‐CI and 21.77% for N‐CI) within three years; 4) enriched genes for potassium‐ion transport and synaptic transmission. Stratification into the two subtypes provides new insight for risk assessment and precise early intervention for MCI patients.

## Graphical abstract

The individual with mild cognitive impairment (MCI) with different subtypes is associated with specific network altered patterns. Two MCI subtypes are identified by employing a regional radiomics similarity network, which shows distinct abnormal patterns in clinical manifestation and clinical outcomes. The stratification into the two subtypes provides a new insight for risk assessment and precise early intervention for MCI patients.

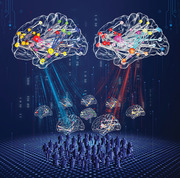

## 1 Introduction

Mild cognitive impairment (MCI) is considered a high‐risk state for developing Alzheimer's disease (AD)^[^ ^1^ ^]^ but has significant phenotypic heterogeneity, both in the clinical presentation^[^ ^2^ ^]^ and in the rate of clinical progression.^[^ ^3^ ^]^ For example, not all subjects with MCI will develop to AD, and some MCI subjects remain stable or even return to normal cognition several years later.^[^ ^3^ ^]^ Thus, recognizing the high‐risk subgroup of MCI at the first visit and understanding how the heterogeneity of MCI influences the subsequent progression to AD or other forms of dementia is crucial for delaying the progression of AD.^[^ ^1^, ^4^ ^]^

Morphological changes in multiple brain regions, particularly in the hippocampus and medial temporal lobe, are potential hallmarks of AD.^[^ ^5^ ^]^ As a transitional stage between a cognitively normal state and AD, MCI patients exhibit pathological features and brain morphological changes similar to those of preclinical AD patients.^[^ ^6^ ^]^ Voxel‐based brain morphometry of MCI patients shows significant heterogeneity, which is associated with the differences in cognitive decline.^[^ ^7^ ^]^ In addition, heterogeneous cortex thickness and a longitudinal progression pattern of cortex thickness in MCI have been reported in previous studies.^[^ ^4^, ^8^ ^]^ Hence, several studies have suggested that the high‐risk/low‐risk subgroups of MCI could be redefined according to brain morphology.

Most previous studies have focused on alterations in the brains of AD patients based only on isolated anatomical regions^[^ ^9^ ^]^ and did not take into account the potential associations with other brain regions.^[^ ^10^ ^]^ It is well accepted that the brain is a complex network. Therefore, evaluating disease‐associated coalterations among distinct anatomical regions opens a new avenue for understanding AD pathology.^[^ ^11^ ^]^ Radiomics features can provide comprehensive and sensitive information about brain regions.^[^ ^12^ ^]^ A regional radiomics similarity network (R2SN) is a novel morphological covariation network with high robustness, stability, and a biological basis.^[^ ^14^ ^]^ R2SN can reflect imperceptible changes in the brain and provide a new perspective for understanding the human brain based on structural T1‐weighted magnetic resonance imaging (sMRI) data. Therefore, based on the R2SN, we hypothesized that clustering analysis could yield distinct MCI subtypes, which would be associated with unique patterns of clinical manifestation abnormalities and longitudinal progression. Furthermore, genetic factors play an important role in AD, so it is crucial to clarify which genetic factors are associated with MCI subtypes.

**Figure 1:**
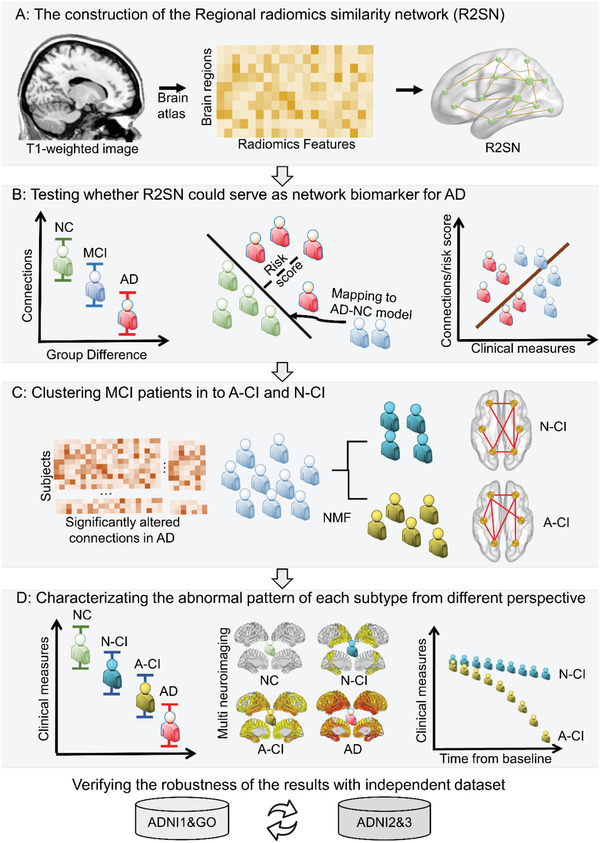
Schematic of the data analysis pipeline. A) The data were preprocessed, and the intensity and textural features were then extracted based on the Brainnetome Atlas. Finally, the network was achieved by computing the Pearson correlation coefficient of each pairwise brain region. B) Difference and classification analysis among the NC, MCI, and AD groups and correlation analysis between risk score/R2SN connections and clinical measures. C) Clustering the MCI group into two different subtypes with NMF. D) Analysis of the subtypes, including clinical measures (cognitive ability, genetic risk, ADAS‐cog, CSF A*β*, and FDG), neuroimaging (R2SN, GM, A*β* PET, and FDG PET), and progression (survival curve, conversion time/rate, and longitudinal change in clinical measures), between the A‐CI and N‐CI groups. Abbreviations: ADNI: Alzheimer's Disease Neuroimaging Initiative; NMF: nonnegative matrix factorization; A*β*: amyloid‐beta; FDG: fluorodeoxyglucose; GMV: gray matter volume; ADAS‐cog: Alzheimer's Disease Assessment Scale–Cognitive Subscale; CSF: cerebrospinal fluid.

We speculated that one subtype of MCI would be similar to normal control (NC) (N‐CI) and that the other subtype of MCI would be similar to AD (A‐CI). We evaluated the differences in the clinical and neuroimaging measures and longitudinal progression patterns between subtypes. Finally, we assessed whether the distinct gene expression profiles were associated with differences in spatial patterns between MCI subtypes (**Figure** **1**).

## 2 Results

### 2.1 Demographic and Neuropsychological Characteristics

The mean age and sex proportion were significantly different (*p* < 0.001) among the NC, MCI, and AD groups. The clinical measures (mini‐mental‐state examination (MMSE) score, polygenic hazard score (PHS), fludeoxyglucose (FDG), Alzheimer's disease assessment scale–cognitive subscale (ADAS‐cog11 score and ADAS‐cog13 score), cerebrospinal fluid (CSF) amyloid‐beta (A*β*) level, CSF Tau level, CSF P‐tau level, cognitive domain composite scores, and auditory‐verbal learning test (AVLT) score) were significantly different among the NC, MCI, and AD groups (*p* < 0.001 with Analysis of Variance (ANOVA), Bonferroni corrected) (**Table** **1**).

**Table 1:** Detailed information on the subjects included in this study

	Group	Age [years]	Sex (M/F)	Clinical measure
	NC (605)	73.47 ± 6.16	279/326	29.08 ± 1.10
Subjects with an MMSE score	MCI (766)	72.96 ± 7.69	450/316	27.57 ± 1.81
(*N* = 1654)	AD (283)	74.91 ± 7.70	152/131	23.18 ± 2.14
	*p*	0.002	<0.001	<0.001
	NC (361)	74.77 ± 5.73	188/173	0.05 ± 0.66
Subjects with a PHS	MCI (632)	73.13 ± 7.53	373/259	0.48 ± 0.81
(*N* = 1228)	AD (235)	75.01 ± 7.55	127/108	0.81 ± 0.84
	*p*	<0.001	0.08	<0.001
	NC (293)	73.88 ± 6.12	152/141	1.31 ± 0.11
Subjects with a FDG measurement	MCI (570)	72.58 ± 7.62	330/240	1.24 ± 0.13
(*N* = 1054)	AD (191)	74.82 ± 7.75	110/81	1.07 ± 0.14
	*p*	<0.001	0.22	<0.001
	NC (210)	74.09 ± 6.04	103/107	1036.81 ± 390.17
Subjects with an A*β* measurement	MCI (421)	72.65 ± 7.48	253/168	843.02 ± 351.84
(*N* = 794)	AD (163)	74.66 ± 7.78	90/73	623.33 ± 245.03
	*p*	0.003	0.03	<0.001
	NC (278)	73.93 ± 6.06	138/140	239.69 ± 91.41
Subjects with a Tau score	MCI (479)	72.40 ± 7.61	281/198	285.05 ± 125.64
(*N* = 926)	AD (169)	74.68 ± 7.76	94/75	368.29 ± 138.86
	*p*	<0.001	0.06	<0.001
	NC (277)	73.95 ± 6.06	137/140	22.13 ± 9.49
Subjects with a P‐tau score	MCI (479)	72.40 ± 7.61	281/198	27.70 ± 14.27
(*N* = 925)	AD (169)	74.68 ± 7.76	94/75	36.88 ± 15.44
	*p*	<0.001	0.05	<0.001
	NC (603)	73.49 ± 6.15	278/325	7.00 ± 3.04
Subjects with an ADAS‐cog11	MCI (765)	72.98 ± 7.68	449/316	10.41 ± 4.42
(*N* = 1650)	AD (282)	74.88 ± 7.70	151/131	19.65 ± 6.66
	*p*	0.002	<0.001	<0.001
	NC (602)	73.51 ± 6.15	278/324	10.38 ± 4.37
Subjects with an ADAS‐cog13 score	MCI (762)	72.97 ± 7.69	448/314	16.64 ± 6.66
(*N* = 1642)	AD (278)	74.93 ± 7.66	148/130	30.03 ± 7.91
	*p*	0.002	<0.001	<0.001
	NC (603)	73.46 ± 6.17	278/325	45.34 ± 9.95
Subjects with an AVLT1 score	MCI (766)	72.96 ± 7.69	450/316	34.52 ± 10.76
(*N* = 1649)	AD (280)	74.83 ± 7.69	149/131	23.09 ± 7.54
	*p*	0.003	<0.001	<0.001
	NC (600)	73.46 ± 6.17	276/324	6.06 ± 2.36
Subjects with an AVLT2 score	MCI (731)	72.85 ± 7.69	428/303	4.26 ± 2.50
(*N* = 1575)	AD (244)	74.7 ± 7.65	131/113	2.0 ± 1.74
	*p*	0.006	<0.001	<0.001

### 2.2 Distinguishing among AD, MCI, and NC in the R2SN

**Figure 2:**
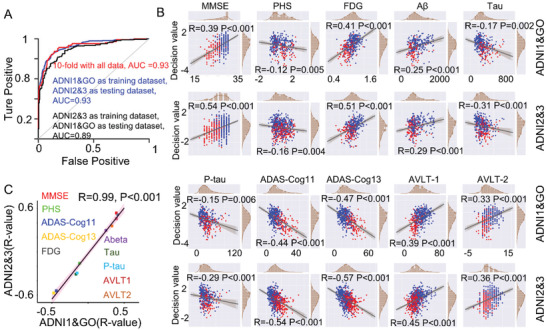
Classification results. A) The AUC for AD versus NC for the tenfold cross‐validation as well as the split sample validation with ADNI1&GO as the training dataset and ADNI2&3 as the testing data, and vice versa. B) The correlation results between the SVM decision value and clinical measures (red: AD, blue: MCI) in the ADNI1&GO dataset and the ADNI2&3 dataset. C) Consistency of the *R*‐values obtained for the correlation between clinical information and decision values in the ADNI1&GO and ADNI2&3 datasets.

Compared with NCs, altered morphological connectivity was found in AD (detailed results are provided in Section S04 in the Supporting Information). Support vector machine (SVM) group separation of AD and NC showed an area under the curve (AUC) = 0.93 (accuracy (ACC) = 0.88, sensibility (SEN) = 0.77, specificity (SPE) = 0.93) using tenfold cross‐validation. We obtained an AUC of 0.93 (ACC = 0.89, SEN = 0.78, SPE = 0.92) when the Alzheimer's Disease Neuroimaging Initiative (ADNI)1&GO subset was used as the training data and ADNI2&3 was used as the testing data. When the ADNI2&3 data were used as the training data, and an AUC = 0.89 (ACC = 0.82, SEN = 0.71, SPE = 0.92) was achieved for the ADNI1&GO used as testing data (**Figure** **2**A). The SVM decision values showed a significant correlation with clinical measures (Figure 2B). These correlations were highly consistent between the ADNI1&GO and ADNI2&3 datasets (Figure 2C) (*R* = 0.99, *p* < 0.001) (Section S04, Supporting Information).

R2SN connections associated with the bilateral hippocampi were significantly correlated with cognitive scores, including the MMSE and AVLT scores. In addition, connections associated with the hippocampus were significantly correlated with the PHS score and ADAS‐cog scores (ADAS‐cog11, ADAS‐cog13). These connections were also significantly correlated with CSF A*β*, CSF Tau, CSF P‐tau, and FDG values (*p* < 0.05, Bonferroni corrected) (Section S04, Supporting Information).

### 2.3 The Different Patterns between MCI Subtypes

#### 2.3.1 Differences in Clinical Information between the Two Subtypes

**Figure 3:**
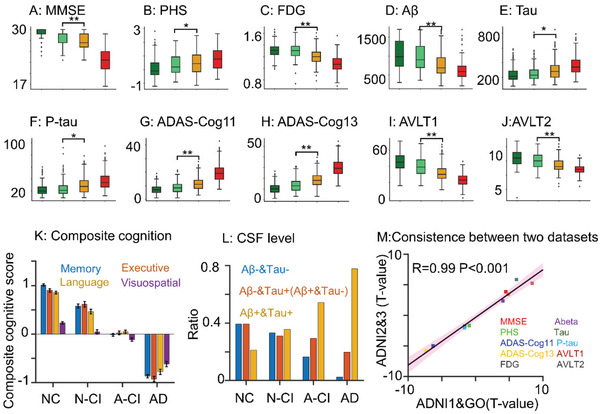
Analysis of the clinical profiles of the diagnostic groups and A‐CI and N‐CI subtypes. A) MMSE score, B) PHS score, C) FDG, D) CSF A*β* level, E) CSF Tau level, F) CSF P‐tau level, G) ADAS‐cog11 score, H) ADAS‐cog13 score, I) AVLT1 score, J) AVLT2 score. K) The cognitive domain composite scores, L) the proportion of CSF A*β*+&Tau+, CSF A*β*−&Tau+/A*β*+&Tau−, and CSF A*β*−&Tau− across the NC, N‐CI, A‐CI, and AD groups. M) The correlation between the *T*‐value of the difference in clinical information between the A‐CI and N‐CI groups in the ADNI1&GO and ADNI2&3 datasets. * *p* < 0.05, ** *p* < 0.001.

The 766 MCI patients were clustered into two subtypes, A‐CI (*N* = 514) and N‐CI (*N* = 252). Patients in the N‐CI group (68.64 ± 7.32) were significantly younger than those in the A‐CI group (75.09 ± 6.95) (*p* < 0.001) (Section S05, Supporting Information). Significant differences between the two groups were also found for MMSE, PHS, AVLT2, AVLT1, ADAS‐cog11, and ADAS‐cog13 scores; CSF A*β*, CSF Tau, and CSF P‐tau levels; and FDG values. All these differences were characterized by *p* < 0.001, except for PHS (*p* = 0.02), CSF Tau (*p* = 0.03), and CSF P‐tau (*p* = 0.01) (**Figure** **3**A–J). The robustness of the result was further tested by a permutation test (1000 permutations), yielding *p* < 0.05 for all comparisons (Section S06, Supporting Information). The cognitive domain composite scores (executive, memory, language, and visuospatial ability) of the A‐CI group were significantly lower than those of the N‐CI group (Figure 3K). In addition, the proportion of A*β*+&Tau+ in the A‐CI group was higher than that in the N‐CI group, and the proportion of A*β*−&Tau− in the A‐CI group was lower than that in the N‐CI group (*p* < 0.05) (Figure 3L). More importantly, a high level of consistency was found for the differences in clinical measures between A‐CI and N‐CI in the ADNI1&GO and ADNI2&3 datasets (R = 0.99, p < 0.001) (Figure 3M). Herein, A‐CI and N‐CI seem to represent different stages of MCI. Thus, we chose a subset of patients from the A‐CI group who showed a similar distribution of MMSE as a subset of patients from the N‐CI group (*p* = 0.85). Interestingly, the progression pattern also showed a significant difference between the subset of A‐CI and N‐CI patients (*p* = 8.22e−7) (Section S07, Supporting Information). Thus, we considered that A‐CI and N‐CI are different subtypes rather than different stages of MCI.

#### 2.3.2 Differences in Neuroimaging Indices between the Two Subtypes

**Figure 4:**
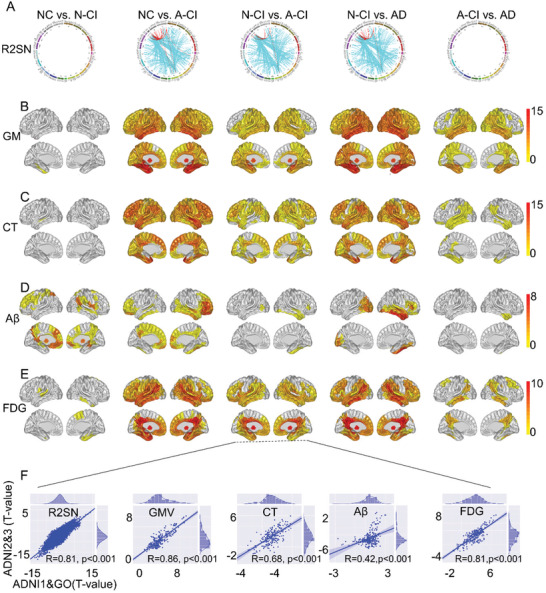
The differences in multimodal neuroimaging indices. A) Differences in the R2SN in NC versus A‐CI, NC versus N‐CI, N‐CI versus A‐CI, A‐CI versus AD, and N‐CI versus AD comparisons; “red” indicates decreased connections, and “blue” indicates increased connections. B) Differences in the GM volume for the pairwise comparisons. The color bar indicates the *T*‐value. C) Differences in the CT value. D) Differences in the A*β* value; the color bar indicates the −1 × *T*‐value. E) Difference in the FDG value; the color bar indicates the *T*‐value. F) The correlation between the *T*‐value of the difference in image indices between A‐CI and N‐CI in the ADNI1&GO and ADNI2&3 datasets.

Significant differences in the hippocampus, temporal lobe, parahippocampal gyrus, and amygdala of the R2SN were found between N‐CI and A‐CI (*p* < 0.05, Bonferroni corrected) (**Figure** **4**A). Additionally, the gray matter (GM) volume, cortex thickness (CT), and FDG of the medial temporal lobe and hippocampus significantly differed between the N‐CI and A‐CI groups (*p* < 0.05, Bonferroni corrected) (Figure 4B,C,E), as was the A*β* of the medial temporal lobe (*p* < 0.05) (Figure 4D). More importantly, a high level of consistency was found for the differences in R2SN, GM, CT, A*β*, and FDG between A‐CI and N‐CI in ADNI1&GO and ADNI2&3 (all *R* > 0.42, *p* < 0.001) (Figure 4F). Further details can be found in Section S08 (Supporting Information).

#### 2.3.3 Differences in Longitudinal Progression between A‐CI and N‐CI

**Figure 5:**
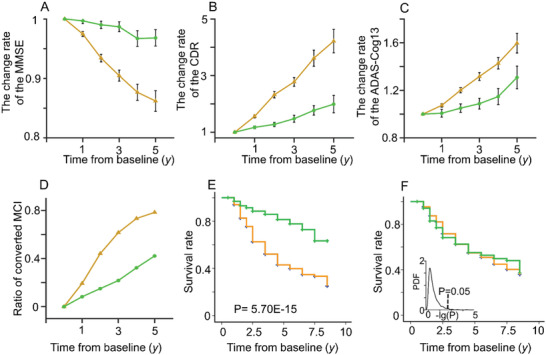
Progression rates in the A‐CI (yellow) and N‐CI (green) groups. The change rate of A) the MMSE, B) CDR, and C) ADAS‐cog13 scores within a 6‐year follow‐up period. D) The proportion of patients with MCI who developed AD in the 6‐year follow‐up period. E) The survival rate of the A‐CI group (*N* = 514) and N‐CI group (*N* = 252). F) Displacement test of the survival rate with randomly reranked labels and the distribution of *p* value.

The MMSE score (**Figure** **5**A), clinical dementia rating (CDR) (Figure 5B), and ADAS‐cog13 score (Figure 5C) showed different patterns in longitudinal progression between the A‐CI and N‐CI groups after age and sex effects were controlled. The ratio of convert/nonconvert patients was also significantly different between the A‐CI and N‐CI groups (*p* < 0.001). The subjects in the A‐CI group had an approximately threefold increased risk of converting to AD than those in the N‐CI group (61.54% of the A‐CI group (*N* = 200/325), and 21.77% of the N‐CI (*N* = 27/124) group were converted within three years) (Figure 5D).

Survival analysis confirmed that individuals in the A‐CI group had a higher risk of conversion to AD and a lower survival rate than those in the N‐CI group (*p* = 5.7e−15) (Figure 5E). The patients in the N‐CI group were significantly younger than those in the A‐CI group (*p* = 2.42e−5). We also recomputed the *p*‐value of the survival analysis by reranking the labels of A‐CI and N‐CI with 1000 random permutations (Figure 5F).

#### 2.3.4 Anatomical Changes in MCI Subtype and Gene Expression Profiling

**Figure 6:**
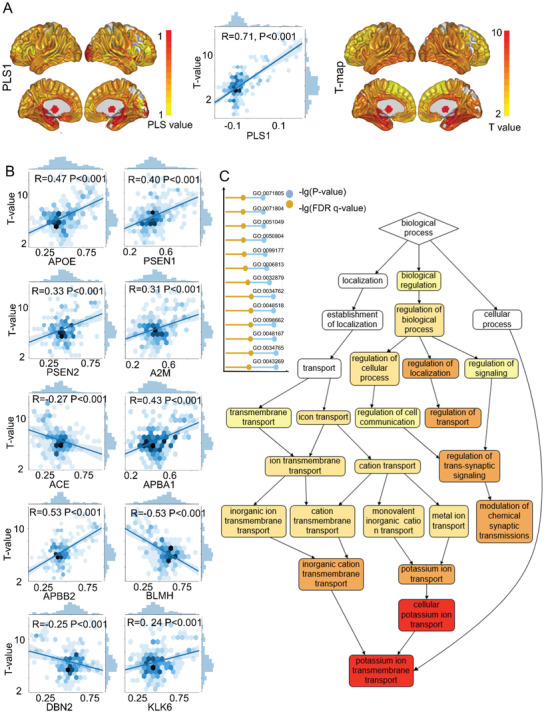
Results of gene set enrichment analysis. A) The weighted regional gene expression of the PLS1 score and the *T*‐map of the difference between A‐CI and N‐CI and the correlation between PLS1 and the *T*‐map of the difference between A‐CI and N‐CI. B) The correlation between the AD‐related genes and the *T*‐map of the difference between A‐CI and N‐CI. C) GO terms showed significance in gene enrichment analysis (*p* < 1e−4, FDR corrected), and the gene pathway diagram summarizing the functional role of the 15 633 genes ranked according to PLS weight. The detailed information about each GO term can be searched in http://geneontology.org/.

The partial least square (PLS) method can estimate the correlation between the *T*‐map of the difference between the A‐CI and N‐CI (independent variable) and regional gene expression values (dependent variables). The first PLS components (PLS1) explained 35% of the variance in the gene expression variables. The PLS1 had significant correlations with the *T*‐map of the difference between the A‐CI and N‐CI groups (*R* = 0.71, *p* < 0.001) (**Figure** **6**A). Furthermore, the 13 AD‐related genes were significantly correlated with the *T*‐map of the difference between the A‐CI and N‐CI groups (*p* < 0.05, Bonferroni corrected), excluding Amyloid Beta (A4) Precursor Protein (APP), Beta‐Site APP‐Cleaving Enzyme 2 (BACE2), and Plasminogen activator urinary (PLAU) (*p* = 0.04, Figure 6B). Gene set enrichment analysis showed that the typical Gene Ontology (GO) terms of biological processes were significantly enriched (false discovery rate correction (FDR) *q*‐value < 1e−5) in potassium‐ion transport (GO: 0006813, FDR *q*‐value = 4.74e−6); regulation of transsynaptic signaling (GO: 0099177, FDR *q*‐value = 5.26e−6); cellular potassium‐ion transport (GO: 0071804, FDR *q*‐value = 5.33e−6); modulation of chemical synaptic transmission (GO: 0050804, FDR *q*‐value = 5.8e−6); and regulation of transport (GO: 0051049, FDR *q*‐value = 6.47e−6) (Figure 6C).

### 2.4 Reproducibility of the Subtypes for Different Brain Atlases or Parcellation Schemes

**Figure 7:**
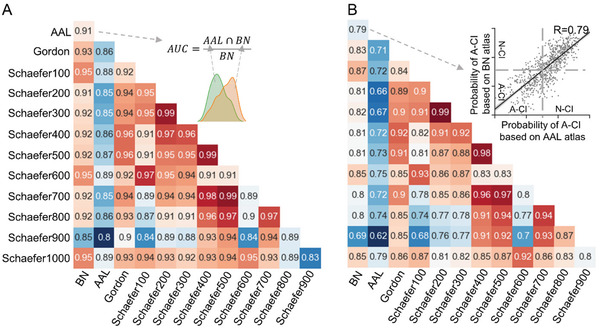
A) The AUC of the overlap of clustering results between any pair of brain atlases. B) The Pearson correlation coefficient of the probability of belonging to the A‐CI group based on different brain atlases.

The MCI subtype was reproducible among the different brain atlases, with all AUCs > 0.8 (**Figure** **7**A). Of these, all AUCs between the subtypes based on the Brainnetome atlas and other brain atlases were >0.9, excluding Schaefer900, which had an AUC = 0.85 (Figure 7A). Meanwhile, the reproducibility of the MCI subtype among different brain atlases was also confirmed, with all *R*‐values > 0.6 (Figure 7B). This result indicates that the MCI subtype is reproducible with different brain atlases.

## 3 Discussion

In this study, we systematically demonstrated that the R2SN could serve as a new network marker for clinical applications in AD/MCI. Two distinct subtypes of the MCI group were identified with a data‐driven method, and the clinical and biological validities of these subtypes were demonstrated. Specifically, the altered pattern of the A‐CI group was consistent with AD, and the N‐CI group was closer to the NCs with respect to R2SN connections, GM volume, metabolic capability, and A*β*. Distinct patterns were associated with the different clinical outcomes of MCI, and two subtypes demonstrated distinct associations with patterns of cognitive impairment and regional gene expression. These results significantly improve our understanding of the heterogeneity in the presentation and clinical outcomes of individuals with MCI.

It is well accepted that the brain is a complex network that supports information transmission.^[^ ^13^ ^]^ The biological basis of this structural covariation network remains controversial, but the large‐scale anatomical covariation network appears to reflect the synchronized maturation or atrophy between pairwise brain regions.^[^ ^11^ ^]^ In addition, R2SN was strongly associated with gene expression and cognitive differences.^[^ ^14^ ^]^ This study also demonstrated that R2SN connections are associated with cognitive ability and clinical biomarker levels. In summary, R2SN can be used to measure the anatomical connectome in vivo and provides a quantitative score of the cognitive impairment of an individual.

Heterogeneous network patterns can provide supporting information for understanding complex brain cognitive functions but have been largely ignored by previous studies;^[^ ^10^, ^11^ ^]^ however, they have begun to be investigated in several recent studies.^[^ ^10^, ^15^ ^]^ The present study, to some extent, remedies the lack of analysis of individual differences in morphological networks among patients with AD. The information transmission capacity of the brain networks will decrease if the stable network structure is broken, which leads to cognitive decline.^[^ ^10^, ^16^ ^]^

Traditional structural covariance networks derived from GM volume or CT have been used to investigate network alterations in brain‐related diseases, including alterations with low sensitivity due to the presence of a single structural biomarker. Thus, to better characterize the coalterations among brain regions, a comprehensive biomarker to estimate the properties of the regions is needed. R2SN is a novel brain covariance network that shows a high association with genes and individual cognitive ability and provides a novel, robust, and biologically plausible model for understanding the human brain. Multivariable classification accuracy was similar to or higher than that of previous studies using a traditional classification model.^[^ ^9^ ^]^ Thus, R2SN is a powerful tool for the study of AD based on structural MRI. To quantitatively estimate the advantage of the R2SN, we also clustered the MCI into two subgroups based on GM volume. However, weaker differences between the two subgroups were obtained when the subgroups were derived based on GM volume (Section S09, Supporting Information). Thus, the abnormal pattern of R2SN is more suitable for investigating the MCI subtype than GM volume.

Converging evidence suggests that individuals with MCI may belong to different subtypes throughout the development of the disease.^[^ ^4a^ ^]^ Not all individuals with MCI will develop AD; some will remain stable or even return to normal cognition several years later.^[^ ^3^ ^]^ MCI is commonly subdivided into amnestic MCI (aMCI) and nonamnestic MCI based on whether memory loss is the dominant cognitive impairment,^[^ ^4^, ^17^ ^]^ with aMCI patients having the highest risk of progression to AD dementia. MCI patients are also commonly stratified into progressive MCI (PMCI) and stable MCI (SMCI) groups. Several studies have suggested that data‐driven cluster analysis based on neuropsychology and clinical presentation seems plausible for identifying the MCI subtype. However, objective subtypes based on neuroimaging studies of MCI have not yet been well established. Our study fills this gap in the field. The patients in the present study were divided into subtypes based only on the phenotype or progression, which offered limited contributions to our understanding of the heterogeneity of MCI. However, the subtypes derived from objective MRI did contribute to our understanding of how MCI is associated with the subsequent progression to AD or other forms of dementia. A greater understanding of how MCI is related to the subsequent progression to AD is beneficial for patient prognosis and the development of precision medicine strategies for MCI.^[^ ^1^, ^4^ ^]^ Thus, two subtypes of MCI (N‐CI and A‐CI) were defined to clarify the relationship among SMCI, PMCI, and AD. We assumed that A‐CI was a high‐risk subtype and that N‐CI was a low‐risk subtype with respect to the conversion to AD dementia. As expected, 61.54% of the A‐CI group and 21.77% of the N‐CI group were converted to AD within three years. The clinical significance of the present study is the identification of high‐risk subjects based on R2SN. A previous study also demonstrated the clinical significance of delaying the conversion to AD in MCI patients.^[^ ^18^ ^]^ The cognition of MCI patients may improve with specific training, as demonstrated in the Mental Activity and Resistance Training trial.^[^ ^19^ ^]^ Our findings suggest that MCI patients in the N‐CI group may receive greater benefits from specific training approaches than those in the A‐CI group, while the A‐CI group may benefit from early clinical intervention/treatment (e.g., cholinesterase inhibitors), as they have already shown AD patterns. However, A‐CI is not equivalent to PMCI, and N‐CI is not equivalent to SMCI. It is well accepted that the conversion of individuals to AD is influenced by multiple factors. Thus, we can only support the conjecture that A‐CI patients have a higher risk of conversion to AD than PMCI patients. More importantly, the A‐CI group showed a faster decline than the N‐CI group when the clinical measures of the two groups were at the same level. We speculate that the abnormal pattern of R2SN can better indicate more advanced disease than clinical measures (such as cognition). Thus, underlying heterogeneity in clinical presentation and progression is critical for patient prognosis and precision medicine strategies for MCI.^[^ ^1^, ^4^ ^]^ Of course, this point should be validated in future studies.

AD is a neurodegenerative disease with multiple genetic risk factors. It is crucial to expound on the genetic significance of A‐CI and N‐CI. Imaging genomics aims to explore the relationship between disease‐related brain regions and genetic risk. Recently, PLS has been successfully used to understand the biological basis of brain connectome changes.^[^ ^20^ ^]^ We found that the anatomical structural changes observed in individuals with A‐CI and N‐CI are associated with changes in gene expression of different brain regions. Furthermore, AD‐related genes were significantly correlated with the changes in anatomical structure observed in A‐CI and N‐CI. This result suggests that the difference in the anatomical structure between A‐CI and N‐CI was consistent with that between AD and NC. Gene set enrichment analysis showed that potassium‐ion transport and regulation of transsynaptic signaling were associated with the *T*‐map of the difference between A‐CI and N‐CI. Therefore, our findings are consistent with the hypothesis that synaptic failure plays an important role in AD.^[^ ^21^ ^]^ Additionally, the concentration of potassium ions is associated with microglial cell activity, which is also considered to play a role in the pathogenesis of AD.^[^ ^22^ ^]^ Brain microglia are crucial for brain health, and they have a dynamic nature and high complexity.^[^ ^23^ ^]^ Brain microglia serve not only as amyloid phagocytes but can also as modulators of neuronal function and homeostasis of the brain.^[^ ^24^ ^]^ However, activated microglia produce several proinflammatory cytokines, which can heighten abnormal protein aggregation and spread.^[^ ^25^ ^]^ A recent study also suggested that microglia release metalloproteases and tau seeds when phagocytosing live tau aggregates.^[^ ^26^ ^]^ In addition, inhibiting microglial proliferation may prevent the progression of Alzheimer's‐like pathology.^[^ ^27^ ^]^ In summary, the evidence suggests that microglia are strongly associated with the pathogenesis of AD, and understanding the relationship between microglia–neuron interactions and brain health is crucial for developing effective therapies for dementia. The results of gene enrichment analysis also confirmed that A‐CI exhibited the same gene pathways as those in AD.

Precision medicine aims to provide personalized treatment strategies by considering disease heterogeneity.^[^ ^28^, ^29^ ^]^ Defining reproducible subtypes is the basis of precision medicine from bench to bedside. In this study, the robustness of the MCI subtypes defined by R2SN was demonstrated by the repetition of the clustered subtypes in different datasets, brain atlases, and/or parcellation schemes. The results also further confirmed the robustness of the radiomics features^[^ ^12^, ^30^ ^]^ and R2SN^[^ ^14^ ^]^ in our previous studies. This study therefore lays a solid foundation for the future development of individualized therapy based on the stratification of R2SN.

Despite these contributions, this study has several limitations. First, the positron emission tomography (PET) images were not included as baseline images due to the limited amount of available data. The robustness of our results should be further explored and validated with additional atlas or independent datasets. In addition, AD was characterized by significant clinical heterogeneity, a crucial confounder for deepening our understanding and enabling more accurate diagnosis, prognosis, and targeted treatment. Furthermore, some mixed factors should be considered in future studies, such as vascular comorbidities, hypertension, and diabetes. Additionally, although the ADNI is a multisite dataset, it has a small number of participants for most of the sites. Thus, we considered the ADNI dataset as a single‐site rather than a multisite dataset. The progression of MCI can lead to many other conditions, such as conversion to NC; thus, more detailed subtypes based on large samples are needed.

## 4 Experimental Section

### Data Acquisition and Clinical Information

A total of 1654 subjects (605 NC, 766 MCI, and 283 AD patients) from the ADNI (http://adni.loni.usc.edu) were included in this study. Informed written consent was obtained from all participants across the ADNI1, ADNIGO, ADNI2, and ADNI3 studies.^[^ ^31^ ^]^ The clinical information included scores from the following assessments: the MMSE, Rey AVLT (including AVLT1: immediate, AVLT2: learning), ADAS‐cog11, ADAS‐cog13, and cognitive domain composite scores, including executive, memory, language, and visuospatial ability. Additionally, CSF A*β*, Tau, and P‐tau values and glucose metabolism derived from FDG PET were obtained. In addition, a PHS for the genetic risk of AD^[^ ^32^ ^]^ was computed from high‐risk genes of AD (Table 1 and Section S01 (Supporting Information)).

### R2SN Construction

For each subject, a T1‐weighted MR image was aligned to Montreal Neurological Institute space using Advanced Normalization Tools (ANTs) and resampled to 1 mm × 1 mm × 1 mm for further analysis. Then, a series of radiomics features (*N* = 47) were extracted for 246 regions defined by the Brainnetome Atlas.^[^ ^33^ ^]^ A common min–max method was first used to normalize the radiomics features among different brain regions in an individual, and the redundancy features were defined as features that had a high correlation with other features (*R* > 0.9).^[^ ^14^ ^]^ As a result, a final feature matrix (246 × 25) for each subject was obtained for further analysis. Briefly, the node of the R2SN was defined as the region based on the Brainnetome Atlas, and the edge was calculated by computing Pearson's correlation coefficient between interregional radiomics features (Figure 1A) (Sections S02 and S03, Supporting Information).^[^ ^14^ ^]^

### The Performance of the R2SN Applied in AD

Whether a R2SN could serve as a neuroimaging biomarker for AD and MCI was first assessed by the following methods. 1) A difference analysis of the R2SN among the NC, MCI, and AD groups was performed. 2) To assess the individual‐prediction performance of the R2SN, a classification model based on a SVM was created. Importantly, to test the robustness of the results, the ADNI dataset was divided into the ADNI1&GO dataset and the ADNI2&3 dataset; one served as training data, and the other served as testing data (and vice versa).^[^ ^5^, ^30^ ^]^ 3) The neurobiological basis of the R2SN was evaluated by relating these connections to other variables, including clinical validity and biological validity (Figure 1B).

### Identifying the Subtypes of MCI

In this study, “consistent” connections were defined as the overlap of the connections obtained from the statistical and classification analyses between the NC and AD groups (Figure 1C).^[^ ^12^ ^]^ MCI patients were clustered into different subtypes using nonnegative matrix factorization (NMF) based on the “consistent” connections. It was speculated that one subtype of MCI would be close to the pattern of NC (N‐CI) and that the other subtype of MCI would be close to the pattern of AD (A‐CI). Hence, the cluster number was set to 2 in the NMF model.

### Differences in the Abnormality Patterns among Subtypes

Characterizing the abnormality pattern of each subtype was one of the crucial steps for understanding MCI. First, to compare the unique attributes of each subtype, the difference in the clinical measures (MMSE, AVLT, PHS, ADAS‐cog11, ADAS‐cog13, CSF A*β*, CSF Tau, CSF P‐tau, and FDG) between the N‐CI and A‐CI groups was assessed with a two‐sample, two‐sided *t*‐test (Figure 1D). To further verify the robustness of the difference between the N‐CI and A‐CI groups, the labels of the subtype were randomly permuted and the difference between the N‐CI and A‐CI groups (1000 permutations) was recalculated. The subjects were divided into three subgroups, A*β*+&Tau+, A*β*−&Tau+/A*β*+&Tau−, and A*β*−&Tau−, in the NC, N‐CI, A‐CI, and AD groups (A*β*+ was defined as A*β* < 980 pg mL^−1^, and Tau+ was defined as Tau > 245 pg mL^−1^, as suggested by the ADNI website https://adni.bitbucket.io/reference/ and https://files.alz.washington.edu/presentations/2018/spring/biomarkers/SHAW.pdf). The chi‐square test was used to determine the statistical significance of the proportion of three CSF biomarker levels. The distribution of the cognitive domain composite score in the NC, N‐CI, A‐CI, and AD groups was also computed.

Then, to investigate the abnormal regions of the brain in each subtype, two‐sample, two‐sided *t*‐tests were performed to evaluate altered regions between groups (NC vs N‐CI, NC vs A‐CI, N‐CI vs A‐CI, AD vs N‐CI, and AD vs A‐CI) based on multimodal neuroimaging indices (R2SN, GM volume, CT, A*β* PET, and FDG PET; all were at the regional level based on the Brainnetome Atlas) (Figure 1D). The GM volume and CT were computed by the CAT12 Toolkit (http://www.neuro.uni‐jena.de/cat/).

Cross‐validation between ADNI1&GO (217 NCs, 453 MCI, and 180 AD) and ADNI2&3 (388 NCs, 313 MCI, and 103 AD) was performed to assess the robustness of the difference between A‐CI and N‐CI. The Pearson correlation coefficients between the results obtained from both datasets were used to estimate the consistency of the findings.^[^ ^12^, ^16^, ^34^ ^]^

### Longitudinal Progression in the A‐CI and N‐CI Groups

The mean values of the abovementioned clinical information were first computed at each annual follow‐up visit to estimate the severity of the decline for each subtype. In addition, survival curves for each subtype were computed with Kaplan–Meier analysis.^[^ ^35^ ^]^ The conversion trajectories were also compared between the N‐CI and A‐CI groups regarding the proportion and timepoints of MCI patients who developed AD within six years (Figure 1D).

### Relationship between the Atrophy Pattern in A‐CI versus N‐CI and Gene Expression Profiling

The potential relationship between gene expression and brain alterations in MCI subtypes was unclear. This relationship was investigated using regional gene expression and maps of altered brain regions in A‐CI compared with N‐CI. Gene expression was initially reported for the Allen atlas (based on 6 NCs) (http://human.brain‐map.org/) and projected to the Brainnetome Atlas using the “abagen” toolkit (https://github.com/rmarkello/abagen). Finally, 15 633 genes from 236 brain regions were obtained. PLS analysis could provide an estimate of the correlation between the metrics^[^ ^36^ ^]^ and was used successfully in previous studies.^[^ ^20^, ^30^ ^]^ Here, the *T*‐map of the GM was used as the independent variable, and the gene expression was used as the dependent variable. The PLS1 was the linear combination of the weighted 15 633 gene expression scores, which were most strongly correlated with the anatomical difference map of MCI subtypes. The enriched GO terms were computed using the “Gorilla” toolkit (http://cbl‐gorilla.cs.technion.ac.il/) after ranking the 15 633 genes with the weighting coefficient obtained from the PLS analysis.

To further determine the relationships between AD‐related gene expression and regional changes in the MCI subtype, 13 AD‐related genes (Alpha‐2‐Macroglobulin (A2M), Angiotensin Converting Enzyme (ACE), Amyloid Beta (A4) Precursor Protein Binding, Family A Member 1 (APBA1), Amyloid Beta Precursor Protein Binding Family B Member 2 (APBB2), Apolipoprotein E (APOE), Amyloid Beta (A4) Precursor Protein (APP), Beta‐Site APP‐Cleaving Enzyme 2 (BACE2), Bleomycin Hydrolase (BLMH), Drebrin 1 (DBN1), Kallikrein‐6 (KLK6), Plasminogen Activator Urinary (PLAU), Presenilin 1 (PSEN1), and Presenilin 2 (PSEN2)) were first identified by searching the disease term “Alzheimer disease” on the AHBA website (https://human.brain‐map.org/microarray/search).^[^ ^37^ ^]^ Then, the Pearson correlation between AD‐related gene expression and the map of altered brain regions was computed for both MCI subtypes.

### The Robustness of the Clustering Results Based on the Different Brain Atlases or Parcellation Schemes

To determine whether the MCI subtypes could be repeated under different brain atlases or parcellation schemes, the Anatomical Automatic Labeling (AAL) Atlas,^[^ ^38^ ^]^ Gordon parcel Atlas,^[^ ^39^ ^]^ and brain parcellation with multiple resolutions from 100 to 1000 parcels^[^ ^40^ ^]^ were studied. The MCI subtype was first defined based on the R2SN in each brain atlas. Then, the AUC of the clustering results and the Pearson correlation coefficient of the probability of belonging to the A‐CI between each pair of brain atlases were used to quantitatively measure the consistency of the subtypes obtained based on different brain atlases or parcellation schemes.

## Conflict of Interest

The authors declare no conflict of interest.

## Supporting information

Supporting Information is available from the Wiley Online Library or from the author.

## Data Availability

The data that support the findings of this study are openly available in Alzheimer's Disease Neuroimaging Initiative at http://adni.loni.usc.edu/, ref. [31].
