# Supplementary material for: Regional Radiomics Similarity Networks Reveal Distinct Subtypes and Abnormality Patterns in Mild Cognitive Impairment
Source: Adv Sci (Weinh). 2022 Jan 31;9(12):2104538. doi: 10.1002/advs.202104538 (PMC9036024; doi:10.1002/advs.202104538)
Supplement: Supplementary file 1 — Supporting Information is available from the Wiley Online Library or from the author. [file ADVS-9-2104538-s001.pdf]

ADVANCED SCIENCE
Open Access

Supporting Information

for *Adv. Sci.,* DOI: 10.1002/advs.202104538

Regional radiomics similarity networks reveal distinct subtypes and abnormality patterns in mild cognitive impairment

Kun Zhao, Qiang Zheng, Martin Dyrba, Timothy Rittman, Ang Li, Tongtong Che, Pindong Chen, Yuqing Sun, Xiaopeng Kang, Qiongling Li, Bing Liu, Yong Liu, Shuyu Li*, for the Alzheimer's Disease Neuroimaging Initiative*

Regional radiomics similarity networks reveal distinct subtypes and abnormality patterns in mild cognitive impairment

S01: Data description

Additional information about the Alzheimer's Disease Neuroimaging Initiative (ADNI) dataset was found in http://adni.loni.usc.edu/wp-content/uploads/how_to_apply/ADNI_Acknowledgement_List.pdf. The clinical information of the baseline was used in this study (https://ida.loni.usc.edu/pages/access/studyData.jsp), and the detailed information about the clinical measures is shown in Table 1.

Data collection and sharing for this project were funded by the Alzheimer’s Disease Neuroimaging Initiative (ADNI) (National Institutes of Health Grant U01 AG024904) and DOD ADNI (Department of Defense award number W81XWH-12-2-0012). ADNI is funded by the National Institute on Aging, the National Institute of Biomedical Imaging and Bioengineering, and generous contributions from AbbVie, Alzheimer’s Association; Alzheimer’s Drug Discovery Foundation; Araclon Biotech; BioClinica, Inc.; Biogen; Bristol-Myers Squibb Company; CereSpir, Inc.; Cogstate; Eisai Inc.; Elan Pharmaceuticals, Inc.; Eli Lilly and Company; EuroImmun; F. Hoffmann-La Roche Ltd, and its affiliated company Genentech, Inc.; Fujirebio; GE Healthcare; IXICO Ltd.; Janssen Alzheimer Immunotherapy Research & Development, LLC.; Johnson & Johnson Pharmaceutical Research & Development LLC.; Lumosity; Lundbeck; Merck & Co., Inc.; Meso Scale Diagnostics, LLC.; NeuroRx Research; Neurotrack Technologies; Novartis Pharmaceuticals Corporation; Pfizer Inc.; Piramal Imaging; Servier; Takeda Pharmaceutical Company; and Transition Therapeutics. The Canadian Institutes of Health Research provide funds to support ADNI clinical sites in Canada. Private sector contributions are facilitated by the Foundation for the National Institutes of Health (www.fnih.org). The grantee organization is the Northern California Institute for Research and Education, and the study is coordinated by the Alzheimer’s Therapeutic Research Institute at the University of Southern California. The ADNI data are disseminated by the Laboratory for Neuro Imaging at the University of Southern California.

The structural MRI (sMRI) data of the baseline was included in this study. The gray matter was segmented based on the cat12 toolkit (http://www.neuro.uni-jena.de/cat/). However, the Aβ PET and the FDG PET data were not completely matched with the sMRI data (not scanning at the same time).

The detailed information about the subject with Aβ PET and the FDG PET is shown in Table S1. More detailed information for data preprocessing of PET images can be found on the ADNI website (http://adni.loni.usc.edu/methods/pet-analysis-method/pet-analysis/#pet-pre-processing-container).

Table S1. The detailed information of the subjects with GM, CT, Aβ PET and FDG PET used in this study

		Age (years)	Sex (M/F)	MMSE
The subjects with GM (N=1654)	NC (605)	73.47±6.16	279/326	29.08±1.10
	N-CI (252)	68.64±7.32	110/142	28.19±1.69
	A-CI (514)	75.09±6.95	340/174	27.26±1.79
	AD (283)	74.91±7.70	152/131	23.18±2.14
The subjects with CT (N=1572)	NC (602)	73.51±6.14	279/323	29.09±1.09
	N-CI (212)	68.96±7.37	95/117	28.13±1.72
	A-CI (475)	75.14±7.01	312/163	27.18±1.79
	AD (283)	74.91±7.70	152/131	23.18±2.14
The subjects with Aβ PET (N=863)	NC (335)	74.25±5.85	173/162	29.07±1.12
	N-CI (191)	68.37±6.88	85/106	28.27±1.61
	A-CI (164)	74.89±6.75	239/125	27.41±1.74
	AD (173)	74.91±7.43	100/73	23.21±2.19
The subjects with FDG PET (N=847)	NC (264)	73.84±5.97	136/128	29.09±1.11
	N-CI (156)	67.90±6.68	73/83	28.26±1.68
	A-CI (282)	74.97±6.68	187/95	27.40±1.74
	AD (145)	74.58±7.63	83/62	23.22±2.19

Note: N-CI and A-CI are derived in the MCI group.

S02: Definitions of the radiomics features

A total of 47 MRI imaging features, including intensity, textural features, were extracted in this study (Aerts et al., 2014). We provided this information in a previous study(Feng et al., 2018; Zhao et al., 2020). To maintain the integrity of this study, we also list it here.

1. Intensity features

Table S2. Intensity features describe the distribution of voxel intensities within an MRI image through commonly used and basic metrics. Because some intensity features have a close relationship with the voxel intensity, such as the maximum and mean, we first normalized those features by the image intensity using the min-max method in each center.

	Image feature	Equation	Definition
Intensity features (14)	Energy	$\sum_{i}^{N} X(i)^2$	Measure of the randomness of the intensity values in an image
	Entropy	$\sum_{i=1}^{N_l} P(i)\log_2 P(i)$	Represents the irregularity of the intensity value distribution
	Kurtosis	$\frac{\frac{1}{N}\sum_{i=1}^{N}(X(i)-\overline{X})^4}{\left(\sqrt{\frac{1}{N}\sum_{i=1}^{N}(X(i)-\overline{X})^2}\right)^2}$	The peakedness of the histogram or indication of histogram flatness
	Maximum	Maximum intensity value of X	
	Mean	$\frac{1}{N}\sum_{i}^{N} X(i)$	Average intensity value of the pixels within the region of interest
	Mean Absolute Deviation (mad)	Mean of the absolute deviations of all voxel intensities around the mean intensity value	A measure of how much the gray levels differ from the mean

Median	Median intensity value of X	
Minimum	Minimum intensity value of X	
Range	Range of intensity values of X	
Root Mean Square (RMS)	$\sqrt{\frac{\sum_{i}^{N} X(i)^{2}}{N}}$	
Skewness	$\frac{\frac{1}{N}\sum_{i=1}^{N}(X(i)-\overline{X})^{3}}{\left(\sqrt{\frac{1}{N}\sum_{i=1}^{N}(X(i)-\overline{X})^{2}}\right)^{3}}$	Symmetry of intensity values in an image
Standard Deviation	$\left(\frac{1}{N-1}\sum_{i=1}^{N}(X(i)-\overline{X})^{2}\right)^{1/2}$	A measure of how much variation or dispersion exists
Uniformity	$\sum_{i=1}^{N_l} P(i)^{2}$	Measures the homogeneity of the intensity value distribution in an image
Variance (Var)	$\frac{1}{N-1}\sum_{i=1}^{N}(X(i)-\overline{X})^{2}$	The spread or variation around the mean (sum of squares)

X denotes the three-dimensional image matrix. ***N*** is the number of voxels. **P** is the first-order histogram with $\boldsymbol{N_l}$ discrete intensity levels. $\overline{X}$ is the mean of X. The number of histogram bins is 100.

2. Textural features

Table S3. Textural features describe the patterns or spatial distribution of voxel intensities.

	Image feature	Equation	Definition
	Autocorrelation	$\sum_{i=1}^{N_g}\sum_{j=1}^{N_g} ij\mathrm{P}(i,j)$	
	Cluster Prominence (CP)	$\sum_{i=1}^{N_g}\sum_{j=1}^{N_g}\left[i+j-\mu_x(i)-\mu_y(j)\right]^4\mathrm{P}(i,j)$	
	Cluster Shade	$\sum_{i=1}^{N_g}\sum_{j=1}^{N_g}\left[i+j-\mu_x(i)-\mu_y(j)\right]^3\mathrm{P}(i,j)$	
	Cluster Tendency	$\sum_{i=1}^{N_g}\sum_{j=1}^{N_g}\left[i+j-\mu_x(i)-\mu_y(j)\right]^2\mathrm{P}(i,j)$	
	Contrast	$\sum_{i=1}^{N_g}\sum_{j=1}^{N_g}\lvert i-j\rvert^2\,\mathrm{P}(i,j)$	Measures the local variation in intensity values
Textural features (33)	Correlation	$\frac{\sum_{i=1}^{N_g}\sum_{j=1}^{N_g} ijP(i,j)-\mu_i(i)\mu_j(j)}{\sigma_x(i)\sigma_y(j)}$	Measures the linear dependencies of intensity values in an image
	Difference Entropy	$\sum_{i=0}^{N_g-1} P_{x-y}(i)log_2\left[P_{x-y}(i)\right]$	
	Dissimilarity	$\sum_{i=1}^{N_g}\sum_{j=1}^{N_g}\lvert i-j\rvert\,\mathrm{P}(i,j)$	
	Energy	$\sum_{i=1}^{N_g}\sum_{j=1}^{N_g}[\mathrm{P}(i,j)]^2$	
	Entropy	$-\sum_{i=1}^{N_g}\sum_{j=1}^{N_g}\mathrm{P}(i,j)\,log_2[\mathrm{P}(i,j)]$	
	Homogeneity1	$\sum_{i=1}^{N_g}\sum_{j=1}^{N_g}\frac{\mathrm{P}(i,j)}{1+\lvert i-j\rvert}$	Measures the homogeneity of the intensity values

Homogeneity2	$\sum_{i=1}^{N_g}\sum_{j=1}^{N_g}\frac{\mathrm{P}(i,j)}{1+\lvert i-j\rvert^2}$	Measures the homogeneity of the intensity values of the pixel pair
Informational Measure of Correlation 1 (IMC1)	$\frac{HXY-HXY1}{max\{HX,HY\}}$	
Informational Measure of Correlation 2 (IMC2)	$\sqrt{1-e^{-2(HXY2-HXY)}}$	
Inverse Difference Moment Normalized (IDMN)	$\sum_{i=1}^{N_g}\sum_{j=1}^{N_g}\frac{\mathrm{P}(i,j)}{1+\left(\frac{\lvert i-j\rvert^2}{N^2}\right)}$	
Inverse Difference Normalized (IDN)	$\sum_{i=1}^{N_g}\sum_{j=1}^{N_g}\frac{\mathrm{P}(i,j)}{1+\left(\frac{\lvert i-j\rvert}{N}\right)}$	
Inverse Variance	$\sum_{i=1}^{N_g}\sum_{j=1}^{N_g}\frac{\mathrm{P}(i,j)}{\lvert i-j\rvert^2}, i\neq j$	
Maximum Probability	$\max\{P(i,j)\}$	
Sum Average	$\sum_{i=2}^{2N_g}\left[i\mathrm{P}_{x+y}(i)\right]$	
Sum Entropy	$-\sum_{i=2}^{2N_g}\mathrm{P}_{x+y}(i)log_2\left[\mathrm{P}_{x+y}(i)\right]$	
Sum Variance	$\sum_{i=2}^{2N_g}(i-SE)^2\,\mathrm{P}_{x+y}(i)$	
Variance	$\sum_{i=1}^{N_g}\sum_{j=1}^{N_g}(i-\mu)^2\,\mathrm{P}(i,j)$	
Short Run Emphasis (SRE)	$\frac{\sum_{i=1}^{N_g}\sum_{j=1}^{N_r}\left[\frac{p(i,j\|\theta)}{j^2}\right]}{\sum_{i=1}^{N_g}\sum_{j=1}^{N_r}p(i,j\|\theta)}$	
Long Run Emphasis (LRE)	$\frac{\sum_{i=1}^{N_g}\sum_{j=1}^{N_r}j^2\,p(i,j\|\theta)}{\sum_{i=1}^{N_g}\sum_{j=1}^{N_r}p(i,j\|\theta)}$	
Gray Level Nonuniformity (GLN)	$\frac{\sum_{i=1}^{N_g}\left[\sum_{j=1}^{N_r}p(i,j\|\theta)\right]^2}{\sum_{i=1}^{N_g}\sum_{j=1}^{N_r}p(i,j\|\theta)}$	Represents the similarity of intensity values in an image

$\mathrm{P}(\mathrm{i},\mathrm{j})$ is the co-occurrence matrix for an arbitrary δ and α

N_g is the number of discrete intensity levels in the image

$p(i,j|\theta)$ is the (i,j)th entry in the given run-length matrix p for a direction θ

N_g is the number of discrete intensity values in the image

N_r is the number of different run lengths

N_p is the number of voxels in the image

u is the mean of $\mathrm{P(i,j)}$

$\mathrm{p_x(i)}=\sum_{j=1}^{N_g}\mathrm{P(i,j)}$ is the marginal row probabilities

$\mathrm{p_y(i)}=\sum_{i=1}^{N_g}\mathrm{P(i,j)}$ is the marginal column probabilities

μ_x is the mean of p_x

μ_y is the mean of p_y

σ_x is the standard deviation of p_x

σ_y is the standard deviation of p_y

$\mathrm{p_{x+y}(k)}=\sum_{i=1}^{N_g}\sum_{j=1}^{N_g}\mathrm{P(i,j)}$, i+j=k, k=2,3,…,2*$N_g$

$\mathrm{P_{x-y}(k)}=\sum_{i=1}^{N_g}\sum_{j=1}^{N_g}\mathrm{P(i,j)}$,$|i-j|$=k, k=0,1,…,$N_g-1$

HX=$-\sum_{i=1}^{N_g}\mathrm{p_x}\,(i)\log_2[\mathrm{p_x(i)}]$ is the entropy of p_x

HY=$-\sum_{i=1}^{N_g}\mathrm{p}_y\,(i)\log_2\left[\mathrm{p_y(i)}\right]$ is the entropy of p_y

H=$-\sum_{i=1}^{N_g}\sum_{j=1}^{N_g}P(i,j)\log_2[P(i,j)]$ is the entropy of $P(i,j)$

HXY1=$-\sum_{i=1}^{N_g}\sum_{j=1}^{N_g}P(i,j)log\left(p_x(i)p_y(j)\right)$

HXY2=$-\sum_{i=1}^{N_g}\sum_{j=1}^{N_g}p_x(i)p_y(j)log\left(p_x(i)p_y(j)\right)$

S03: Supplementary method for R2SN construction

We first performed a common min-max method to normalize the radiomics feature among different brain regions in an individual, and the redundancy feature was defined as those features which had a high correlation with others feature (R>0.9) (Zhao et al., 2021). Those superfluous features were removed before subsequent analysis. As a result, a final feature matrix with 25 × 246 (Brainnetome atlas (Fan et al., 2016)) for each subject was obtained for further analysis. The reserved features are shown in Table S4. The detailed brain regions' names of the Brainnetome atlas are shown in Table S5.

Table S4. The reserved features after removing superfluous features

**Intensity features**	energy
	kurtosis
	maximum
	mad
	minimum
	skewness
	entropy
**Textural features**	Autocorrelation
	Cluster Prominence
	Cluster Shade
	Cluster Tendency
	Contrast
	Correlation
	Energy
	Entropy
	Homogeneity1
	IMC1
	Maximum Probability
	Sum Entropy
	Short Run Emphasis
	Long Run Emphasis
	Gray Level Nonuniformity
	Low Gray Level Run Emphasis
	High Gray Level Run Emphasis
	Long Run High Gray Level Emphasis

Table S5. The detailed brain regions' name of Brainnetome atlas (https://atlas.brainnetome.org/).

Lobe	Gyrus	Left and Right Hemisphere	Label ID.L	Label ID.R	Anatomical and modified Cyto-architectonic descriptions
**Frontal Lobe**	SFG, Superior Frontal Gyrus	SFG_L(R)_7_1	1	2	*A8m, medial area 8*
		SFG_L(R)_7_2	3	4	*A8dl, dorsolateral area 8*
		SFG_L(R)_7_3	5	6	*A9l, lateral area 9*
		SFG_L(R)_7_4	7	8	*A6dl, dorsolateral area 6*
		SFG_L(R)_7_5	9	10	*A6m, medial area 6*
		SFG_L(R)_7_6	11	12	*A9m,medial area 9*
		SFG_L(R)_7_7	13	14	*A10m, medial area 10*
	MFG, Middle Frontal Gyrus	MFG_L(R)_7_1	15	16	*A9/46d, dorsal area 9/46*
		MFG_L(R)_7_2	17	18	*IFJ, inferior frontal junction*
		MFG_L(R)_7_3	19	20	*A46, area 46*
		MFG_L(R)_7_4	21	22	*A9/46v, ventral area 9/46*
		MFG_L(R)_7_5	23	24	*A8vl, ventrolateral area 8*
		MFG_L(R)_7_6	25	26	*A6vl, ventrolateral area 6*
		MFG_L(R)_7_7	27	28	*A10l, lateral area10*
	IFG, Inferior Frontal Gyrus	IFG_L(R)_6_1	29	30	*A44d,dorsal area 44*
		IFG_L(R)_6_2	31	32	*IFS, inferior frontal sulcus*
		IFG_L(R)_6_3	33	34	*A45c, caudal area 45*
		IFG_L(R)_6_4	35	36	*A45r, rostral area 45*
		IFG_L(R)_6_5	37	38	*A44op, opercular area 44*
		IFG_L(R)_6_6	39	40	*A44v, ventral area 44*
	OrG, Orbital Gyrus	OrG_L(R)_6_1	41	42	*A14m, medial area 14*
		OrG_L(R)_6_2	43	44	*A12/47o, orbital area 12/47*
		OrG_L(R)_6_3	45	46	*A11l, lateral area 11*
		OrG_L(R)_6_4	47	48	*A11m, medial area 11*
		OrG_L(R)_6_5	49	50	*A13, area 13*
		OrG_L(R)_6_6	51	52	*A12/47l, lateral area 12/47*
	PrG, Precentral Gyrus	PrG_L(R)_6_1	53	54	*A4hf, area 4(head and face region)*
		PrG_L(R)_6_2	55	56	*A6cdl, caudal dorsolateral area 6*
		PrG_L(R)_6_3	57	58	*A4ul, area 4(upper limb region)*
		PrG_L(R)_6_4	59	60	*A4t, area 4(trunk region)*
		PrG_L(R)_6_5	61	62	*A4tl, area 4(tongue and larynx region)*
		PrG_L(R)_6_6	63	64	*A6cvl, caudal ventrolateral area 6*
	PCL, Paracentral	PCL_L(R)_2_1	65	66	*A1/2/3ll, area1/2/3 (lower limb*

	Lobule				*region)*
		PCL_L(R)_2_2	67	68	*A4ll, area 4, (lower limb region)*
**Temporal Lobe**	STG, Superior Temporal Gyrus	STG_L(R)_6_1	69	70	*A38m, medial area 38*
		STG_L(R)_6_2	71	72	*A41/42, area 41/42*
		STG_L(R)_6_3	73	74	*TE1.0 and TE1.2*
		STG_L(R)_6_4	75	76	*A22c, caudal area 22*
		STG_L(R)_6_5	77	78	*A38l, lateral area 38*
		STG_L(R)_6_6	79	80	*A22r, rostral area 22*
	MTG, Middle Temporal Gyrus	MTG_L(R)_4_1	81	82	*A21c, caudal area 21*
		MTG_L(R)_4_2	83	84	*A21r, rostral area 21*
		MTG_L(R)_4_3	85	86	*A37dl, dorsolateral area37*
		MTG_L(R)_4_4	87	88	*aSTS, anterior superior temporal sulcus*
	ITG, Inferior Temporal Gyrus	ITG_L(R)_7_1	89	90	*A20iv, intermediate ventral area 20*
		ITG_L(R)_7_2	91	92	*A37elv, extreme lateroventral area37*
		ITG_L(R)_7_3	93	94	*A20r, rostral area 20*
		ITG_L(R)_7_4	95	96	*A20il, intermediate lateral area 20*
		ITG_L(R)_7_5	97	98	*A37vl, ventrolateral area 37*
		ITG_L(R)_7_6	99	100	*A20cl, caudolateral of area 20*
		ITG_L(R)_7_7	101	102	*A20cv, caudoventral of area 20*
	FuG, Fusiform Gyrus	FuG_L(R)_3_1	103	104	*A20rv, rostroventral area 20*
		FuG_L(R)_3_2	105	106	*A37mv, medioventral area37*
		FuG_L(R)_3_3	107	108	*A37lv, lateroventral area37*
	PhG, Parahippocampal Gyrus	PhG_L(R)_6_1	109	110	*A35/36r, rostral area 35/36*
		PhG_L(R)_6_2	111	112	*A35/36c, caudal area 35/36*
		PhG_L(R)_6_3	113	114	*TL, area TL (lateral PPHC, posterior parahippocampal gyrus)*
		PhG_L(R)_6_4	115	116	*A28/34, area 28/34 (EC, entorhinal cortex)*
		PhG_L(R)_6_5	117	118	*TI, area TI(temporal agranular insular cortex)*
		PhG_L(R)_6_6	119	120	*TH, area TH (medial PPHC)*
	pSTS, posterior Superior Temporal Sulcus	pSTS_L(R)_2_1	121	122	*rpSTS, rostroposterior superior temporal sulcus*
		pSTS_L(R)_2_2	123	124	*cpSTS, caudoposterior superior temporal sulcus*
**Parietal Lobe**	SPL, Superior Parietal Lobule	SPL_L(R)_5_1	125	126	*A7r, rostral area 7*
		SPL_L(R)_5_2	127	128	*A7c, caudal area 7*

		SPL_L(R)_5_3	129	130	*A5l, lateral area 5*
		SPL_L(R)_5_4	131	132	*A7pc, postcentral area 7*
		SPL_L(R)_5_5	133	134	*A7ip, intraparietal area 7(hIP3)*
	IPL, Inferior Parietal Lobule	IPL_L(R)_6_1	135	136	*A39c, caudal area 39(PGp)*
		IPL_L(R)_6_2	137	138	*A39rd, rostrodorsal area 39(Hip3)*
		IPL_L(R)_6_3	139	140	*A40rd, rostrodorsal area 40(PFt)*
		IPL_L(R)_6_4	141	142	*A40c, caudal area 40(PFm)*
		IPL_L(R)_6_5	143	144	*A39rv, rostroventral area 39(PGa)*
		IPL_L(R)_6_6	145	146	*A40rv, rostroventral area 40(PFop)*
	Pcun, Precuneus	PCun_L(R)_4_1	147	148	*A7m, medial area 7(PEp)*
		PCun_L(R)_4_2	149	150	*A5m, medial area 5(PEm)*
		PCun_L(R)_4_3	151	152	*dmPOS, dorsomedial parietooccipital sulcus(PEr)*
		PCun_L(R)_4_4	153	154	*A31, area 31 (Lc1)*
	PoG, Postcentral Gyrus	PoG_L(R)_4_1	155	156	*A1/2/3ulhf, area 1/2/3(upper limb, head and face region)*
		PoG_L(R)_4_2	157	158	*A1/2/3tonIa, area 1/2/3(tongue and larynx region)*
		PoG_L(R)_4_3	159	160	*A2, area 2*
		PoG_L(R)_4_4	161	162	*A1/2/3tru, area1/2/3(trunk region)*
**Insular Lobe**	INS, Insular Gyrus	INS_L(R)_6_1	163	164	*G, hypergranular insula*
		INS_L(R)_6_2	165	166	*vIa, ventral agranular insula*
		INS_L(R)_6_3	167	168	*dIa, dorsal agranular insula*
		INS_L(R)_6_4	169	170	*vId/vIg, ventral dysgranular and granular insula*
		INS_L(R)_6_5	171	172	*dIg, dorsal granular insula*
		INS_L(R)_6_6	173	174	*dId, dorsal dysgranular insula*
**Limbic Lobe**	CG, Cingulate Gyrus	CG_L(R)_7_1	175	176	*A23d, dorsal area 23*
		CG_L(R)_7_2	177	178	*A24rv, rostroventral area 24*
		CG_L(R)_7_3	179	180	*A32p, pregenual area 32*
		CG_L(R)_7_4	181	182	*A23v, ventral area 23*
		CG_L(R)_7_5	183	184	*A24cd, caudodorsal area 24*
		CG_L(R)_7_6	185	186	*A23c, caudal area 23*
		CG_L(R)_7_7	187	188	*A32sg, subgenual area 32*
**Occipital Lobe**	MVOcC, MedioVentral Occipital Cortex	MVOcC _L(R)_5_1	189	190	*cLinG, caudal lingual gyrus*
		MVOcC _L(R)_5_2	191	192	*rCunG, rostral cuneus gyrus*
		MVOcC _L(R)_5_3	193	194	*cCunG, caudal cuneus gyrus*
		MVOcC _L(R)_5_4	195	196	*rLinG, rostral lingual gyrus*

		MVOcC _L(R)_5_5	197	198	*vmPOS,ventromedial parietooccipital sulcus*
	LOcC, lateral Occipital Cortex	LOcC_L(R)_4_1	199	200	*mOccG, middle occipital gyrus*
		LOcC _L(R)_4_2	201	202	*V5/MT+, area V5/MT+*
		LOcC _L(R)_4_3	203	204	*OPC, occipital polar cortex*
		LOcC_L(R)_4_4	205	206	*iOccG, inferior occipital gyrus*
		LOcC _L(R)_2_1	207	208	*msOccG, medial superior occipital gyrus*
		LOcC _L(R)_2_2	209	210	*lsOccG, lateral superior occipital gyrus*
**Subcortical Nuclei**	Amyg, Amygdala	Amyg_L(R)_2_1	211	212	*mAmyg, medial amygdala*
		Amyg_L(R)_2_2	213	214	*lAmyg, lateral amygdala*
	Hipp, Hippocampus	Hipp_L(R)_2_1	215	216	*rHipp, rostral hippocampus*
		Hipp_L(R)_2_2	217	218	*cHipp, caudal hippocampus*
	BG, Basal Ganglia	BG_L(R)_6_1	219	220	*vCa, ventral caudate*
		BG_L(R)_6_2	221	222	*GP, globus pallidus*
		BG_L(R)_6_3	223	224	*NAC, nucleus accumbens*
		BG_L(R)_6_4	225	226	*vmPu, ventromedial putamen*
		BG_L(R)_6_5	227	228	*dCa, dorsal caudate*
		BG_L(R)_6_6	229	230	*dlPu, dorsolateral putamen*
	Tha, Thalamus	Tha_L(R)_8_1	231	232	*mPFtha, medial pre-frontal thalamus*
		Tha_L(R)_8_2	233	234	*mPMtha, pre-motor thalamus*
		Tha_L(R)_8_3	235	236	*Stha, sensory thalamus*
		Tha_L(R)_8_4	237	238	*rTtha, rostral temporal thalamus*
		Tha_L(R)_8_5	239	240	*PPtha, posterior parietal thalamus*
		Tha_L(R)_8_6	241	242	*Otha, occipital thalamus*
		Tha_L(R)_8_7	243	244	*cTtha, caudal temporal thalamus*
		Tha_L(R)_8_8	245	246	*lPFtha, lateral pre-frontal thalamus*

S04. Estimating the network properties of the R2SN in AD

Statistical analysis and classification

First, the statistical significance of group differences was determined by a two-sample two-sided *t*-test between each pair of two groups (NC vs. MCI, NC vs. AD, and MCI vs. AD). Due to the 30315 comparisons (symmetric matrix with 246×246) for estimating the group difference of the R2SN, a Bonferroni correction with $P < 0.05$ was used to reduce false-positive findings. To further assess the individual-prediction performance of the R2SN, a nonlinear support vector machine (SVM) with a radial basis function (RBF) kernel was trained based on the LIBSVM library (http://www.csie.ntu.edu.tw/~cjlin/libsvm/), as in our previous study. The accuracy (ACC), specificity (SPE), sensitivity (SEN), and area under the receiver operating characteristic curve (AUC) were used to evaluate the performance of the classification model (10-fold cross-validation). To further test the robustness of the SVM classification results, we trained our model on the ADNI1&GO datasets (including 217 NCs, 453 MCI and 180 AD) and validated it on the ADNI2&3 datasets (388 NCs, 313 MCI and 103 AD) and vice versa.

Compared to NCs, there was altered morphological connectivity in AD, especially in the connections associated with the middle and inferior temporal gyrus, inferior, parahippocampal gyrus, cingulate gyrus, amygdala, and hippocampus (Figure S1a-c). The altered morphological connectivity was also evident in MCI, but alterations were weak compared to the alterations present in AD. Notably, some of the connections of the bilateral hippocampi were significantly different in all pairwise group comparisons ($P<0.05$, Bonferroni corrected).

SVM group separation of AD and NC showed an AUC = 0.93 (ACC=0.88, SEN = 0.77, SPE = 0.93) using 10-fold cross-validation. We obtained an ACC of 0.89 (SPE=0.92, SEN=0.78, AUC=0.93) when the ADNI1&GO subset was used as training data and ADNI2&3 as testing data. For training on ADNI2&3, an ACC=0.82 (SPE=0.92, SEN=0.71, AUC=0.89) was achieved for the ADNI1&GO as testing data. The SVM decision values showed a significant correlation with the MMSE score, AVLT score, PHS, CSF Aβ level, CSF Tau level, CSF P-tau level, FDG value, and ADAS-cog score in the AD and MCI groups (all $P<0.05$, Bonferroni corrected) in both datasets ADNI1&GO and ADNI2&3. These correlations were highly consistent between the ADNI1&GO and ADNI2&3 datasets ($R=0.99$, $P<0.001$.

Table S6. The ACC, SEN, SPE and AUC in distinguishing AD from NC based on different kernel function of SVM model.

Kernel function	Training dataset	Testing dataset	ACC (%)	SPE (%)	SEN (%)	AUC
Linear	ADNI1&GO	ADNI2&3	0.88	0.80	0.90	0.92
	ADNI2&3	ADNI1&GO	0.76	0.58	0.90	0.86
RBF	ADNI1&GO	ADNI2&3	0.89	0.78	0.92	0.93
	ADNI2&3	ADNI1&GO	0.82	0.71	0.92	0.89

The associations between R2SN connectivity and clinical measures

We evaluated the neurobiological basis of the R2SN connectivity strength by relating these connections to other variables, including clinical validity (ie, MMSE, AVLT, and ADAS-cog scores), and biological validity (ie, PHS, CSF Aβ levels, and FDG data). Pearson's correlation coefficients were calculated (P<0.05, Bonferroni corrected) between the identified R2SN connections and the abovementioned clinical measures. To verify the clinical relevance of the classification, we also investigated the correlations between the decision score of the SVM model and the abovementioned clinical information of the test subjects in the MCI and AD groups (P<0.05, Bonferroni corrected).

To test the robustness of the correlation results, we evaluated the correlation between R2SN connectivity/decision values and clinical measures in independent ADNI1&GO datasets (including 217 NCs, 453 MCI and 180 AD) and ADNI2&3 datasets (388 NCs, 313 MCI and 103 AD). The R-values obtained for the correlations between clinical measures and decision values from the two datasets were used to estimate the consistency of the results.

R2SN connections associated with the bilateral hippocampi were significantly correlated with cognitive scores, including the MMSE and AVLT scores. In addition, connections associated with the hippocampus were significantly correlated with the PHS score and ADAS-cog scores (ADAS-cog11, ADAS-cog13); moreover, these connections were also significantly correlated with CSF Aβ, CSF Tau, CSF P-tau and FDG values (P<0.05, Bonferroni corrected) (Figure S1d-m).

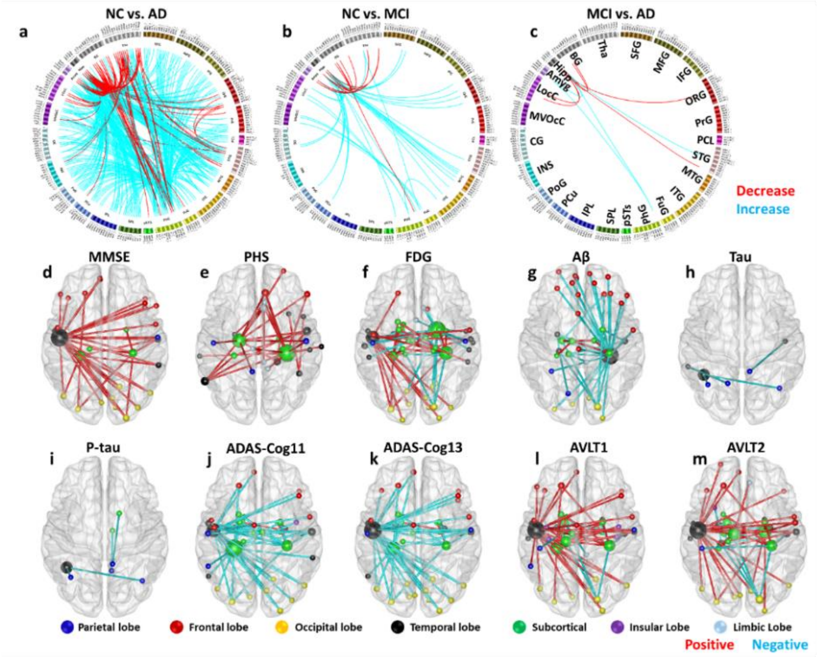

Figure S1. The statistical results of the R2SN. Group differences for (a) NC vs. AD, (b) NC vs. MCI, and (c) MCI vs. AD. R2SN connections correlated with clinical measures for (d) MMSE, (e) PHS, (f) FDG, (g) CSF Aβ, (h) CSF Tau, (i) CSF P-tau, (j) ADAS-Cog11, (k) ADAS-Cog13, (l) AVLT1, and (m) AVLT2. For (d-m), we only show the 60 most significant connections.

S05: Supplementary result for the clinical information in A-CI and N-CI

Fifty "consistent" connections showed significant differences between the AD and NC groups, meanwhile showed significant contribution in classification AD from NC. At last, the 50 consistent connections were used to cluster the subtype of the MCI (Figure S2).

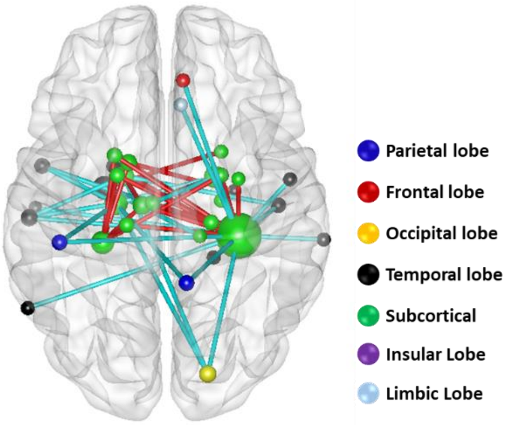

Figure S2. The R2SN connections are used on the clustering subtype of the MCI.

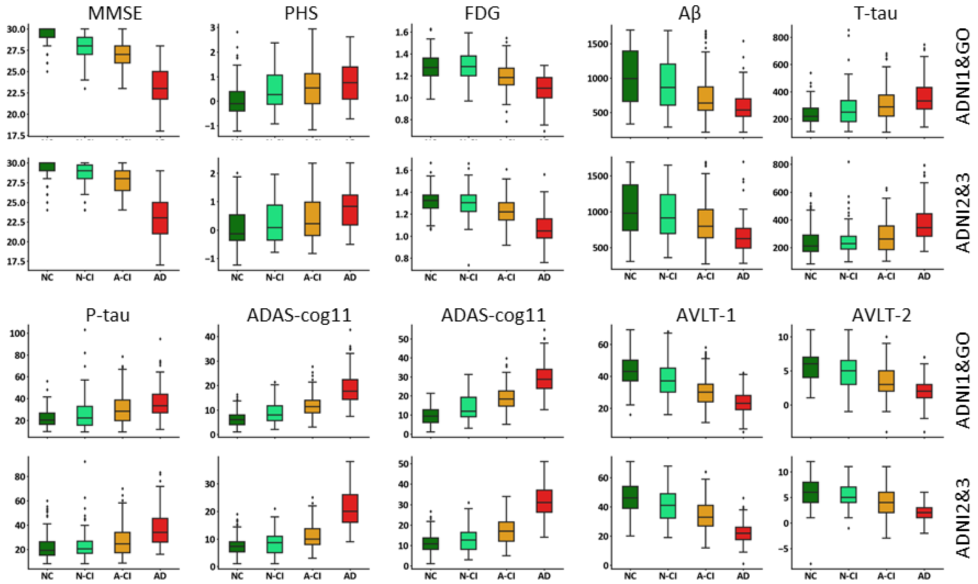

Figure S3. Analysis of the clinical profiles of the diagnostic groups and A-CI and N-CI subtypes. (A) MMSE score, (B) PHS score, (C) FDG, (D) CSF Aβ level (E) CSF Tau level, (F) CSF P-tau level, (G) ADAS-Cog11 score, (H) ADAS-Cog13 score, (I) AVLT1 score, (J) AVLT2 score.

We computed the clinical information in the A-CI and N-CI. Interesting, the age of the A-CI was significantly older than that in the N-CI (Table S7).

Table S7. The detailed clinical information in N-CI and A-CI

	Group	Age(years)	Sex(M/F)	Clinical measures
Subjects with MMSE	A-CI(514)	75.09±6.95	340/174	27.26±1.79
	N-CI(252)	68.64±7.32	110/142	28.19±1.69
	P	<0.001	<0.001	<0.001
Subjects with PHS	A-CI(445)	75.11±6.83	294/151	0.52±0.83
	N-CI(187)	68.42±7.04	79/108	0.37±0.73
	P	<0.001	<0.001	0.027
Subjects with ADAS11	A-CI(514)	75.09±6.95	340/174	11.3±4.29
	N-CI(251)	68.67±7.32	109/142	8.59±4.11
	P	<0.001	<0.001	<0.001
Subjects with ADAS13	A-CI(511)	75.08±6.95	339/172	18.25±6.28
	N-CI(251)	68.67±7.32	109/142	13.37±6.20
	P	<0.001	<0.001	<0.001
Subjects with Aβ	A-CI(289)	74.65±6.66	198/91	784.61±331.33
	N-CI(132)	68.29±7.36	55/77	970.9±362.68
	P	<0.001	<0.001	0.02
Subjects with FDG	A-CI(350)	75.00±6.75	231/119	1.20±0.12
	N-CI(220)	68.75±7.35	99/121	1.30±0.12
	P	<0.001	<0.001	<0.001
Subjects with Tau	A-CI(313)	74.71±6.78	211/102	298.95±127.24
	N-CI(166)	68.04±7.19	70/96	258.85±118.57
	P	<0.001	<0.001	<0.001
Subjects with P-tau	A-CI(313)	74.71±6.78	211/102	29.38±14.52
	N-CI(166)	68.04±7.19	70/96	24.54±13.27
	P	<0.001	<0.001	<0.001
Subjects with AVLT1	A-CI(514)	75.09±6.95	340/174	31.72±9.33
	N-CI(252)	68.64±7.32	110/142	40.23±11.24
	P	<0.001	<0.001	<0.001
Subjects with AVLT2	A-CI(486)	75.00±6.95	321/165	3.76±2.39
	N-CI(245)	68.58±7.31	107/138	5.26±2.41
	P	<0.001	<0.001	<0.001

S06: Supplementary result for the permutation test of the difference between A-CI and N-CI

MCI is a heterogeneous group. Thus, we speculated that the different result between A-CI and N-CI was caused by the influence of subtype, rather than the variation of MCI subjects.

To verify the robustness of the result, we re-labeled the subtype (the number of subjects for each subtype remained the same) by absolutely random and computed the difference of clinical information between N-CI and A-CI (Table S8).

As expected, we found that little difference can be obtained if we re-label the subtype with absolutely random. Thus, we speculated that the different clinical findings between A-CI and N-CI are objective existed.

Table S8. The detailed results for the P-value for difference analysis and permutation test between N-CI and A-CI.

	P value1	P value2
MMSE	$<10^{-16}$	0
PHS	0.024	0.024
ADAS-Cog11	6.35×10^{-7}	0
ADAS-Cog13	5.22×10^{-11}	0
FDG	$<10^{-16}$	0
Aβ	5.21×10^{-8}	0
Tau	0.03	0.025
P-Tau	0.01	0.013
AVLT1	$<10^{-16}$	0
AVLT2	2.49×10^{-14}	0

P value1: the P-value of difference analysis between A-CI and N-CI.

P value2: the P-value of permutation test between A-CI and N-CI, in brief, the subtype label was randomly re-ranked with 1000 times, and the P value2 = times (P< P value1)/1000.

S07: Supplementary result for the progression of the subtypes

In this study, 514 individuals with A-CI and 252 individuals with N-CI were obtained. The MMSE and other clinical measures showed a significant difference between A-CI and N-CI. It seems to be a stage rather than a subtype. Thus, we chose a subset of the A-CI who showed a similar pattern of MMSE as N-CI (P=0.85). Interestingly, the progression pattern also showed a significant difference between the subset of A-CI and N-CI (P=8.22E-7) (Figure S4). Thus, we considered that the A-CI and N-CI are the different subtypes rather than the different stages of the MCI.

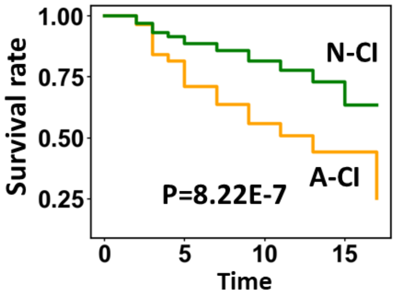

Figure S4. The longitudinal progression of the subset of A-CI and N-CI.

S08: Supplementary result for the neuroimaging data in NC, N-CI, A-CI and AD

Significant differences in the hippocampus, temporal lobe, parahippocampal gyrus, amygdala of the R2SN were found between N-CI and A-CI ($P<0.05$, Bonferroni corrected). Additionally, the GM volume, CT and the FDG of the medial temporal lobe and hippocampus significantly differed between N-CI and A-CI ($P<0.05$, Bonferroni corrected); and Aβ of the medial temporal lobe was significantly different between N-CI and A-CI ($P<0.05$). More importantly, a high consistency was obtained between the difference of R2SN, GM, CT, Aβ, and FDG between A-CI and N-CI in ADNI1&GO and ADNI2&3 (all $R>0.42$, $P<0.001$).

The connections associated with the hippocampi bilaterally showed significant differences between the A-CI and NC groups ($P<0.05$, Bonferroni corrected), and the abnormal pattern of A-CI was similar to that of AD. However, the connections associated with the anterior central lobule and the temporal lobe were significantly different between the N-CI and NC groups. The GM volume, CT and FDG value of the medial temporal lobe, hippocampus, gyrus cingula, and precuneus showed significant differences between the A-CI and NC groups ($P<0.05$, Bonferroni corrected). Interestingly, GM volume, CT and the FDG value showed little difference between the NC and N-CI groups. The Aβ of the medial temporal lobe was significantly different between the A-CI and NC groups, and the Aβ showed less of a difference between the N-CI and NC groups than between the A-CI and NC groups ($P<0.05$).

The connections associated with the bilateral caudate showed significant differences between the A-CI and AD groups ($P<0.05$), and the connections associated with the bilateral hippocampi and parietal lobe were significantly different between the N-CI and AD groups ($P<0.05$). GM volumes and CT of the medial temporal lobe, hippocampus, gyrus cingula, precuneus, and frontal lobe showed significant differences between the N-CI and AD groups ($P<0.05$, Bonferroni corrected). Also, GM volume and CT showed differences between the A-CI and AD groups ($P<0.05$, Bonferroni corrected) but the differences were weaker than those between the N-CI and AD groups. The Aβ value of the medial temporal lobe was significantly different between the N-CI and AD groups, and Aβ values showed little difference between the A-CI and AD groups ($P<0.05$). FDG values of the medial temporal lobe, hippocampus, gyrus cingula, precuneus, and frontal lobe showed significant differences between the N-CI and AD groups ($P<0.05$, Bonferroni corrected), and FDG values of part of the medial temporal lobe and of the precuneus showed weaker differences between the A-CI and AD groups than between the N-CI and AD groups ($P<0.05$, Bonferroni corrected).

S09: Supplementary result for the subtyping result based on gray matter volume

To verify the advantage of the current study, we also computed the subtyping result based on the R2SN and gray matter (GM) volume, respectively. Weaker differences were obtained in the subtype of GM than the subtype of R2SN. Detailed results are shown in Table S9.

Table S9. The P-value of the difference between N-CI and A-CI is based on R2SN and GM volume, respectively.

	P (R2SN, $N_{N\text{-}CI}$=252, $N_{A\text{-}CI}$=514)	P (GM, $N_{N\text{-}CI}$=675, $N_{A\text{-}CI}$=92)
MMSE	$<10^{-16}$	9.80×10^{-6}
PHS	0.024	0.92
ADAS-Cog11	6.35×10^{-7}	0.13
ADAS-Cog13	5.22×10^{-11}	0.10
FDG	$<10^{-16}$	1×10^{-4}
Aβ	5.21×10^{-8}	0.21
Tau	0.03	0.008
P-Tau	0.01	0.008
AVLT1	$<10^{-16}$	0.007
AVLT2	2.49×10^{-14}	0.04

Reference

Aerts HJ, Velazquez ER, Leijenaar RT, Parmar C, Grossmann P, Carvalho S*, et al.* Decoding tumour phenotype by noninvasive imaging using a quantitative radiomics approach. Nat Commun 2014; 5: 4006.

Fan L, Li H, Zhuo J, Zhang Y, Wang J, Chen L*, et al.* The Human Brainnetome Atlas: A New Brain Atlas Based on Connectional Architecture. Cereb Cortex 2016; 26(8): 3508-26.

Feng F, Wang P, Zhao K, Zhou B, Yao H, Meng Q*, et al.* Radiomic Features of Hippocampal Subregions in Alzheimer's Disease and Amnestic Mild Cognitive Impairment. Front Aging Neurosci 2018; 10: 290.

Zhao K, Ding YH, Han Y, Fan Y, Alexander-Bloch AF, Han T*, et al.* Independent and reproducible hippocampal radiomic biomarkers for multisite Alzheimer's disease: diagnosis, longitudinal progress and biological basis. Science Bulletin 2020; 65(13): 1103-13.

Zhao K, Zheng Q, Che T, Dyrba M, Li Q, Ding Y*, et al.* Regional radiomics similarity networks (R2SNs) in the human brain: reproducibility, small-world properties and a biological basis. Network Neuroscience 2021: 1-30.
